# Ag(I) camphorimine complexes with antimicrobial activity towards clinically important bacteria and species of the *Candida* genus

**DOI:** 10.1371/journal.pone.0177355

**Published:** 2017-05-09

**Authors:** João M. S. Cardoso, Soraia I. Guerreiro, Andreia Lourenço, Marta M. Alves, M. Fátima Montemor, Nuno P. Mira, Jorge H. Leitão, M. Fernanda N. N. Carvalho

**Affiliations:** 1 Centro de Química Estrutural, Instituto Superior Técnico, Universidade de Lisboa, Av. Rovisco Pais Lisboa, Portugal; 2 iBB—Institute for Bioengineering and Biosciences and Department of Bioengineering, Instituto Superior Técnico, Universidade de Lisboa, Av. Rovisco Pais, Lisboa, Portugal; Natural Environment Research Council, UNITED KINGDOM

## Abstract

The present work follows a previous report describing the antibacterial activity of silver camphorimine complexes of general formula [Ag(NO_3_)L]. The synthesis and demonstration of the antifungal and antibacterial activity of three novel [Ag(NO_3_)L] complexes (named **1**, **2** and **3**) is herein demonstrated. This work also shows for the first time that the previously studied complexes (named **4** to **8**) also exert antifungal activity. The antibacterial activity of complexes was evaluated against *Staphylococcus aureus*, *Pseudomonas aeruginosa*, *Burkholderia contaminans* and *Escherichia coli* strains, while antifungal activity was tested against the *Candida* species *C*. *albicans*, *C*. *glabrata*, *C*. *parapsilosis* and *C*. *tropicalis*. The antimicrobial activity of the complexes ranged from very high (complex **4**) to moderate (complex **6**) or low (complex **8**), depending on the structural and electronic characteristics of the camphorimine ligands. Notably, the highest antibacterial and anti-*Candida* activities do not coincide in the same complex and in some cases they were even opposite, as is the case of complex **4** which exhibits a high anti-bacterial and low antifungal activity. These distinct results suggest that the complexes may have different mechanisms against prokaryotic and eukaryotic cells. The antifungal activity of the Ag(I) camphorimine complexes (in particular of complex **1**) was found to be very high (MIC = 2 μg/mL) against *C*. *parapsilosis*, being also registered a prominent activity against *C*. *tropicalis* and *C*. *glabrata*. None of the tested compounds inhibited *C*. *albicans* growth, being this attributed to the ability of these yeast cells to mediate the formation of less toxic Ag nanoparticles, as confirmed by Scanning Electron Microscopy images. The high antibacterial and anti-*Candida* activities of the here studied camphorimine complexes, especially of complexes **1** and **7**, suggests a potential therapeutic application for these compounds.

## Introduction

An increasing number of pathogenic bacterial and fungal strains resistant to existing antibiotics and antifungals have been isolated either from hospitals or the community. This situation is a threat for public health since existing pharmaceuticals become non-effective and in some cases useless to treat infections.

In the case of bacteria, the problem of resistance to antibiotics is particularly severe among members of the so-called ESKAPE group that includes *Enterococcus faecium*, *Staphylococcus aureus*, *Klebsiella pneumoniae*, *Acinetobacter baumannii*, *Pseudomonas aeruginosa* and Enterobacteriaceae [1]. In the case of fungi, the main concerns come from an increasing resistance of the *Candida* species (namely, *C*. *albicans*, *C*. *glabrata*, *C*. *tropicalis* and *C*. *parapsilosis*) to the clinically used antifungals [2,3]. Infections caused by the above-mentioned bacterial and fungal species are emerging and are responsible for high rates of mortality and morbidity, posing serious challenges to the sustainability of healthcare systems [4]. In this scenario, the identification of new compounds with efficacy against emerging pathogens for the development of more effective therapies [5] is a challenge to the academic community and an opportunity to the development of alternative products by the pharmaceutical industry. Silver nitrate and silver sulphadiazine have been efficiently used as components of balms to prevent infections on wounds or burns [6,7]. However, their therapeutic use as antimicrobials has been limited by toxicity at concentrations higher than 1% [8–10]. Albeit the toxicity has to be considered, the emergent potential of silver based species (compounds [11–13] or nanoparticles [14–18]) as antimicrobials challenge the design, synthesis, trial and assessment of the properties of new silver compounds against bacteria and fungi to explore their therapeutic and antiseptic properties (e.g. in water purification properties or on coating of implants surface [19–22]).

In the present study, we focused on the synthesis and evaluation of the antibacterial and antifungal properties of silver camphorimine complexes obtained from silver nitrate and camphor derivatives. The choice of camphor as a precursor for ligands to synthesize new complexes comes from the fact that such as silver nitrate camphor has a long history of pharmacological uses, *e*.*g*. as anesthetic, muscular relaxant or insect repellent, either the compound itself or as part of natural products [23–25]. Information on the antimicrobial applications of camphor are scarce, although studies showed that camphorimine derivatives have antiviral properties against HIV [26] or influenza virus [27]. In addition, previous results obtained by us on the assessment of the antibacterial activity of camphorimine Ag(I) complexes showed that some have MIC (minimum inhibitory concentration) values considerably lower than AgNO_3_ [28]. In this work we have investigated the activity of the three newly synthesized camphorimine Ag(I) complexes **1**–**3** against the ESKAPE members *S*. *aureus*, *P*. *aeruginosa*, and *E*. *coli*, the *B*. *contaminans* IST408 isolate from a Portuguese cystic fibrosis patient [29, 30]. We also report results on the antifungal activity of the Ag(I) camphorimine complexes **1**–**8** against the *Candida* species *C*. *albicans*, *C*. *glabrata*, *C*. *parapsilopsis* and *C*. *tropicalis*.

## Materials and methods

### Chemicals and chemical techniques

All the complexes were synthesized under inert gas atmosphere using vacuum / Schlenk techniques. The camphor derivatives were synthesized under air using conventional organic synthesis techniques. Complexes (**4**–**8**) [28,31] and ligands (L^II^, L^IV^-L^VIII^) [32,33] were synthesized following published procedures. Camphor, camphorsulfonic acid and the appropriate amines were purchased from Sigma-Aldrich (St. Louis, MO, USA). The solvents were of PA grade and were pre-dried (using the suitable drying agent) [34] and distilled under nitrogen immediately before use. Acetonitrile was from Carlo Erba (France) and THF and all the other solvents from Panreac (Germany). The IR spectra were obtained from KBr pellets using a JASCO FT/IR 4100 spectrometer. NMR spectra (^1^H, ^13^C, DEPT and HSQC, HMBC) were obtained from chloroform-d_1_ or acetonitrile-d_3_ solutions using Bruker Avance II^+^ (300 or 400 MHz) spectrometers. NMR chemical shifts are referred to TMS (δ = 0 ppm). Cyclic voltammograms (CV) were obtained from Bu_4_NBF_4_/CH_3_CN (0.10 M) solutions using a three compartments cell, equipped with platinum wire working and secondary electrodes interfaced with a VoltaLab PST050 equipment. The potentials were measured in Volts (± 10 mV) *versus* SCE at 200 mV/s using (Fe(η^5^-C_5_H_5_)_2_]^0/+^ (*E*_1/2_ = 0.382 V) as internal reference. Elemental analysis was based on the complete oxidation of the sample (flash combustion) in a EA 1108 CHNS-O Element Analyser from Thermo Scientific (Fisons) (Waltham, MA, USA). The resulting combustion gases passed through a reducing furnace and swept into the chromatographic column by helium (carrier gas), where they were separated and quantitatively detected by appropriated gas analysis procedures using a TCD detector.

### Ligands synthesis

(1,2)-1,2-bis((1,4)-1,7,7-trimethylbicyclo[2.2.1]heptan-2-ylidene)hydrazine, (**L**^**I**^)—(1*R*)-(+)-Camphor (610 mg; 4.0 mmol) was stirred in etanol/acetic acid (10/0.5 mL) for *ca*. 1h at RT. Then, hydrazine monohydrate (0.1 mL; 2.0 mmol) was added and the mixture stirred for 72 h at 50°C. Yield 55%. Elem. Anal. (%) for C_20_H_32_N_2_ ½H_2_O: Found: C, 77.6; N, 9.3; H, 10.7. Calc.: C, 77.7; N, 9.1; H, 10.7. for IR (cm^-1^): 1670. ^1^H NMR: (CDCl_3_, δ ppm): 2.28 (d, *J* = 18 Hz, 2H), 1.97–1.77 (m, 6H), 1.71 (t, *J* = 12.0 Hz, 2H), 1.47 (t, *J* = 12.0 Hz, 2H), 1.22 (t, *J* = 12.0 Hz, 2H), 1.04 (s, 6H), 0.92 (s, 6H), 0.78 (s, 6H). ^13^C NMR: (CDCl_3_, δ ppm): 176.2 (C2), 52.8 (C1), 48.0 (C7), 43.9 (C4), 35.4 (C3), 32.6 (C6), 27.2 (C5), 19.6, 18.8, (C9, C10), 11.2 (C8).

(3,3)-3,3'-([1,1'-biphenyl]-4,4'-diylbis(azanylylidene))bis(1,7,7-trimethylbicyclo [2.2.1] heptan-2-one), (**L**^**III**^)—Camphorquinone (500 mg; 3.0 mmol) was stirred in EtOH (10 mL) acidified with acetic acid (0.3 mL) at room temperature for 30 minutes. Benzidine (275 mg; 1.5 mmol) was then added and the yellow mixture was stirred at 50°C for 18 hours. After cooling, the solvent was removed under vacuum and the yellow solid was washed with Et_2_O and *n*-pentane. Yield 86%. Elem. Anal. (%) for C_32_H_36_N_2_O_2_: Found: C, 80.0; N, 5.7; H, 7.8. Calc.: C, 80.0; N, 5.8; H, 7.6. IR (cm^-1^): 1745 (ν_CO_), 1661 (ν_CN_). ^1^H NMR: (CDCl_3_, δ ppm): 7.59 (d, *J* = 8.0 Hz, 4H), 7.03 (d, *J* = 8.0 Hz, 4H), 2.90 (d, *J* = 4.0 Hz, 2H), 2.15–2.11 (m, 2H), 1.90–1.86 (m, 2H), 1.73–1.63 (m, 4H), 1.12 (s, 6H), 1.00 (s, 6H), 0.93 (s, 6H). ^13^C NMR: (CDCl_3_, δ ppm): 206.7 (C2), 172.1 (C3), 148.8 (C_*ipso*_), 137.7 (C_*ipso*_), 127.5 (C_Ph_), 121.4 (C_Ph_), 58.1 (C1), 50.4 (C4), 44.8 (C7), 30.3 (C6), 24.6 (C5), 21.2, 17.7, (C9, C10), 9.3 (C8).

### Complexes synthesis

[Ag(NO_3_)(C_20_H_32_N_2_)] (**1**)–AgNO_3_ (85 mg, 0.5 mmol) and compound **L**^**I**^ (150 mg, 1.0 mmol) were mixed and stirred under vacuum for 30 minutes. THF (10 mL) was then added and the solution was stirred for 72 hours at 40°C. The solvent was fully evaporated and the off-white solid obtained was recrystallized from CH_2_Cl_2_ / Et_2_O. A white solid was obtained which was filtered off solution and dried under vacuum. Yield 84%. Elem. Anal. (%) for AgC_20_H_32_N_3_O_3_.(½CH_2_Cl_2_): Found: C, 47.6; N, 8.5; H, 6.7. Calc.: C, 48.0; N, 8.2; H, 6.5. IR (cm^-1^): 1655 (υ_CN_); 1385 (υ_NO3_). ^1^H NMR (CD_3_CN, δ ppm): 2.17 (s, 4H), 1.88–1.77 (m, 6H), 1.41–1.35 (m, 2H), 1.24–1.18 (m, 2H), 1.01 (s, 6H), 0.93 (s, 6H), 0.76 (s, 6H). ^13^C NMR (CD_3_CN, δ ppm): 175.0 (C2), 53.2 (C1), 48.7 (C7), 44.8 (C4), 36.0 (C3), 33.4 (C6), 27.9 (C5), 19.9, 19.0 (C9, C10), 11.7 (C8).

[Ag(NO_3_)(C_26_H_32_N_2_O_2_)] (**2**)–AgNO_3_ (170 mg, 1.0 mmol) and **L**^**II**^ (405 mg, 1.0 mmol) were mixed and stirred under vacuum for 30 minutes. Then, acetonitrile (10 mL) was added and the solution stirred at room temperature for 18 hours. A yellow solid formed that was filtered off solution and washed with Et_2_O and *n*-pentane. Yield 86%. Elem. Anal. (%) for AgC_26_H_32_N_3_O_5_.H_2_O: Found: C, 52.6; N, 7.0; H, 5.5. Calc.: C, 52.7; N, 7.1; H, 5.8. IR (cm^-1^): 1748 (υ_CO_); 1661 (υ_CN_); 1384 (υ_NO3_). ^1^H NMR (CD_3_CN, δppm): 7.42 (t, *J* = 8 Hz, 1H), 6.79 (d, *J* = 8 Hz, 2H), 6.29 (s, 1H), 2.80 (d, *J* = 4 Hz, 2H), 2.12–2.06 (m, 2H), 1.96–190 (m, 2H), 1.63–1.51 (m, 4H), 1.05 (s, 6H), 0.99 (s, 6H), 0.84 (s, 6H). ^13^C NMR (CD_3_CN, δppm): 206.7 (C2), 174.9 (C3), 151.0 (C_*ipso*_), 131.2 (C_Ph_), 118.7 (C_Ph_), 113.0 (C_Ph_), 59.0 (C1), 51.4 (C4), 45.4 (C7), 30.8 (C6), 24.4 (C5), 21.3, 17.4 (C9, C10), 9.3 (C8).

[Ag(NO_3_)(C_32_H_36_N_2_O_2_)] (**3**)–AgNO_3_ (170 mg, 1.0 mmol) and **L**^**III**^ (480 mg, 1.0 mmol) were mixed and stirred under vacuum for 30 minutes. Acetonitrile (10 mL) was then added and the solution was stirred at room temperature for 18 hours. The yellow suspension was filtered off solution and the solid was washed with *n*-pentane. Yield 81%. Elem. Anal. (%) for AgC_32_H_36_N_3_O_5_ ½H_2_O: Found: C, 58.1; N, 6.8; H, 5.6. Calc.: C, 58.3; N, 6.4; H, 5.7. IR (cm^-1^): 1746 (υ_CO_); 1662 (υ_CN_); 1384 (υ_NO3_). ^1^H NMR (CD_3_CN, δppm): 7.68 (d, *J* = 8 Hz, 4H), 7.04 (d, *J* = 8 Hz, 4H), 2.87 (d, *J* = 4 Hz, 2H), 2.17–2.11 (m, 2H), 1.97–191 (m, 2H), 1.70–1.58 (m, 4H), 1.07 (s, 6H), 1.00 (s, 6H), 0.87 (s, 6H). ^13^C NMR (CD_3_CN, δppm): 207.2 (C2), 173.8 (C3), 149.7 (C_*ipso*_), 138.5 (C_*ipso*_), 128.4 (C_Ph_), 122.3 (C_Ph_), 58.9 (C1), 51.4 (C4), 45.4 (C7), 30.9 (C6), 24.8 (C5), 21.2, 17.5 (C9, C10), 9.3 (C8).

### Bacterial strains and assessment of antibacterial activity

When in use, *Staphylococcus aureus* Newman, *Pseudomonas aeruginosa* 477, *Escherichia coli* ATCC 25922, and *Burkholderia contaminans* IST408 were routinely maintained in Lennox Broth (LB) solid medium (Sigma-Aldrich, St. Louis, USA). The antimicrobial activity of the camphorimine Ag(I) complexes were quantified by estimating the MIC based on standard methods [35] as previously described [36]. Briefly, the MIC values of the compounds were determined as follows: overnight grown bacterial cultures (carried out in Mueller-Hinton (MH) broth (Sigma-Aldrich, St. Louis, USA) at 37°C and 250 rev.min^-1^) were diluted with fresh MH medium to a final optical density of 0.02, measured at 640 nm (OD_640_) in a Hitachi U-2000 UV/Vis spectrophotometer. From these cell suspensions, 100 μL aliquots were mixed in the wells of 96-wells polystyrene plates with 100 μL of fresh MH supplemented with the compounds under study, previously serially diluted (1:2) from stock solutions of each compound. After incubation for 24 h at 37°C, the OD_640_ of the cultures were measured using a Spectrostar^Nano^ microplate reader (BMG Labtech, Germany).

The MIC values were estimated by fitting the OD_640_ mean values with a Gompertz modified equation as described before [28]. Mean OD_640_ values were obtained from a total of at least 4 experiments from 2 independently prepared MICs assays performed as duplicates. Colony-forming units (CFUs) of the bacterial strains in cultures carried out in the presence of concentrations of the compounds below and above the estimated MIC values were assessed by spot inoculating onto the surface of LB solid medium 10 μL aliquots of serially diluted samples.

### *Candida* strains and assessment of antifungal activity

The ability of the camphor-derived Ag(I) complexes (**1**–**8**) to inhibit the growth of *C*. *albicans*, *C*. *glabrata*, *C*. *tropicalis* and *C*. *parapsilosis* was assessed using the standardized microdilution method recommended by EUCAST (European Committee on Antimicrobial Susceptibility Testing) to determine the MIC_50_ values, considered to be the concentration of drug that reduced yeast growth by more than 50% the growth registered in drug-free medium [37]. The strains used in this work were *C*. *albicans* SC5314, *C*. *glabrata* CBS138, *C*. *parapsilosis* ATCC 22019 and *C*. *tropicalis* ATCC750. Briefly, cells of the different species were cultivated (at 30°C and with 250 rpm orbital agitation) for 17h in YPD growth medium and then diluted in fresh RPMI growth medium (Sigma) to obtain a cell suspension having an OD_530nm_ of 0.05. From these cell suspensions, 100 μL aliquots were mixed in the 96-multiwell polystyrene plates with 100 μL of fresh RPMI medium (control) or with 100 μL of this same medium supplemented with 2000, 1000, 500, 250, 125, 62.5, 31.3, 15.63, 7.81 and 3.91 μg/mL of the different compounds. As a control we have also examined the inhibitory effect of AgNO_3_ and that of the free ligands. After inoculation, the 96-multiwell plates were incubated without agitation at 37°C for 24h. After that time, cells were re-suspended and the OD_530nm_ of the cultures was measured in a microplate reader. The MIC_50_ value was taken as being the highest concentration tested at which the growth of the strains was 50% of the value registered in the control lane.

### Microscopic characterization of complexes and cells

The physico-chemical characterization of the complexes and the cells before and after incubation in the presence of the complexes was performed using scanning electron microscopy (SEM) with a JEOL-JSM7001F apparatus. The elemental chemical composition was performed by the respective X-ray energy dispersive spectrometer (EDS). To increase the conductivity of the samples they were coated with a thin layer of conductive chromium (Polaron E-5100). The histograms for the diameter distributions of the nanoparticles were produced from SEM images using ImageJ software.

## Results and discussion

### Synthesis and characterization of the compounds

**Fig 1:**
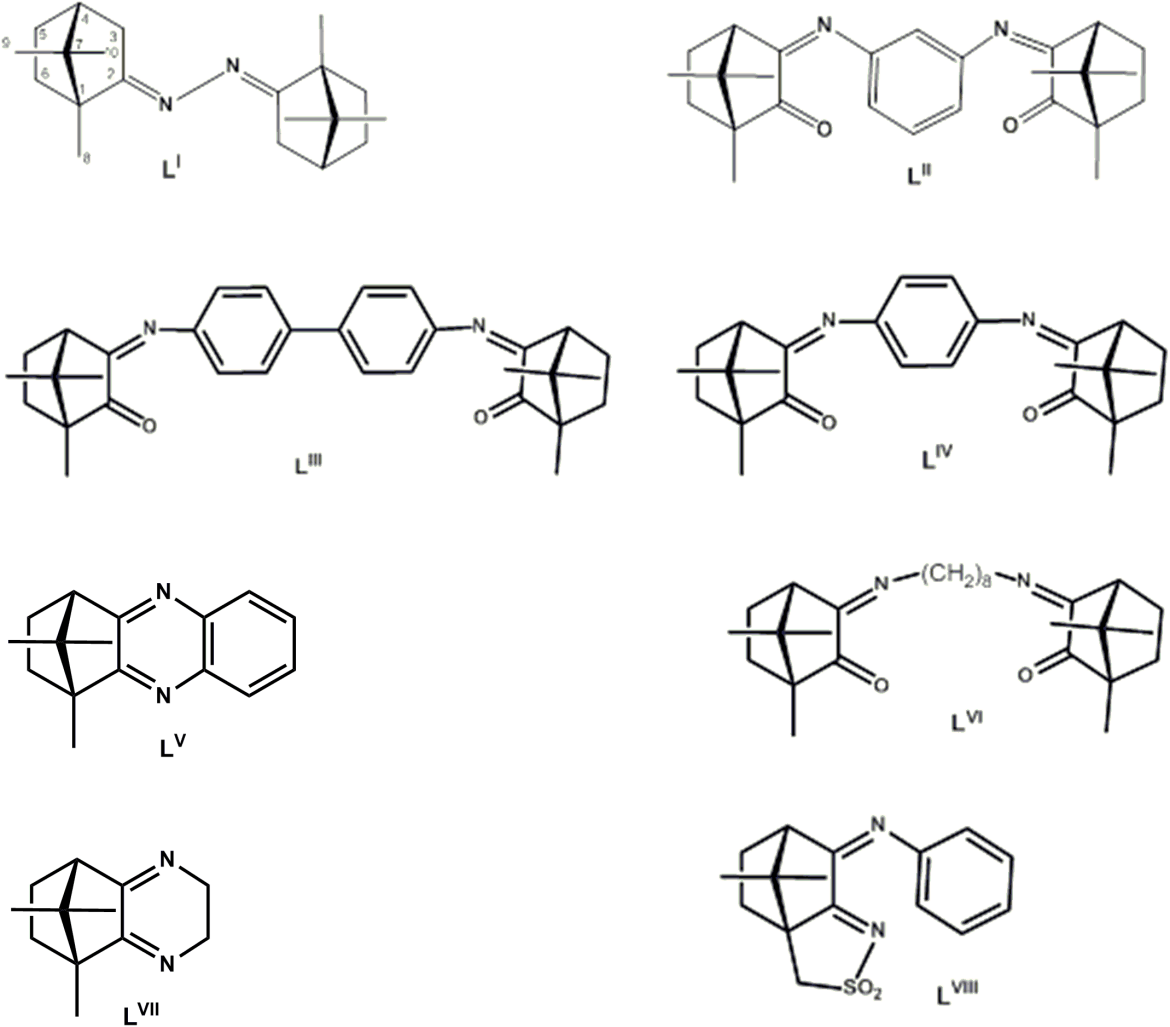
Camphorimine compounds used as ligands.

Functionalization of camphor or camphorquinone by condensation with primary amines or hydrazines is a strategy typically used to synthesize compounds that essentially keep the bicyclic structure of camphor with improved ability to coordinate transition metals. This way, several camphorimine derivatives were prepared which were used as ligands (Fig 1) to coordinate silver nitrate. The camphor derivatives **L**^**I**^ and **L**^**III**^ are new, while the other camphor compounds (**L**^**II**^, **L**^**IV**^**—L**^**VIII**^) were previously reported [28]. The two new compounds were formulated based on analytical and spectroscopic data (FTIR and NMR). Formulation by elemental analysis is corroborated by observation of just one band in the region assigned to the stretching of the CN and CO double bonds (1750–1600 cm^-1^) in the IR spectrum of **L**^**I**^ (ν_CN_, 1670 cm^-1^) while in the IR spectrum of **L**^**III**^ two bands (ν_CN_ 1661 cm^-1^; ν_CO_ 1745cm^-1^) are observed, as expected. The NMR spectra evidence in **L**^**I**^, **L**^**III**^ equivalent camphor skeletons and imine (**L**^**I**^, 176.2 ppm, C2; **L**^**III**^, 172.1 ppm, C3) and ketone (**L**^**III**^, 206.7 ppm, C2) groups with the right camphor/aromatic linker integration consistent with formulation.

By reaction of the appropriate camphor derivative (**L**) with AgNO_3_, three new complexes [Ag(NO_3_)L] (L = **L**^**I**^, **1**; **L**^**II**^, **2**; **L**^**III**^, **3**) were synthesized that were characterized by elemental analysis, FTIR and NMR. The most relevant differences in the spectra of the complexes and the corresponding ligands rely on a band attributable to the NO_3_ stretching (1384–1385 cm^-1^) observed in the IR spectra of the complexes and absent in the spectra of the ligands.

**Fig 2:**
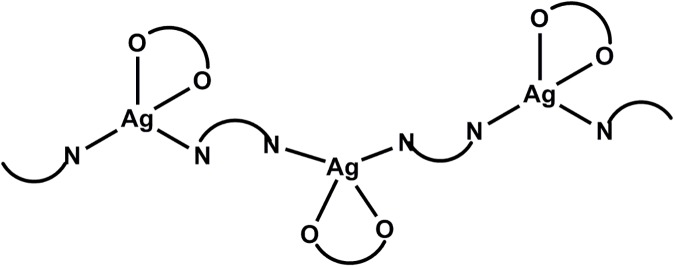
Structural arrangement of compounds [Ag(NO_3_)L] (L = N͡N), according to crystallographic data obtained for 5 [28].

The new compounds (**1**–**3**) enlarge the variety of the [Ag(NO_3_)L]_n_ camphorimine complexes previously synthesized from the ligands **L**^**IV**^- **L**^**VIII**^ (**4**–**8**), essentially keeping their formulation and structure according to elemental analysis and spectroscopic data, *i*.*e*. the camphorimine ligands binding the silver metal through the nitrogen atom of the camphor imine group and the NO_3_ group through two oxygen atoms within a zig-zag polymeric arrangement (Fig 2) as found by X-ray analysis for **5** [28].

#### Redox properties

The redox properties of the complexes and of the ligands were evaluated by cyclic voltammetry. The complexes display one irreversible cathodic process at a potential close to that of AgNO_3_ reduction (0.18 V) attributed to Ag(I)→Ag(0) reduction (Table 1). Additionally, reversible cathodic and anodic waves at potentials in the range of those of the corresponding free camphorimine compounds (Ligand) are indicative of ligand based processes (Table 1). Adsorption waves, detected on the reverse scan upon reduction, evidence deposition of Ag(0) on the working electrode, thus corroborating silver metal formation upon electron transfer. The potential range of the cathodic process (**1**–**3**; 0.049 to 0.14 V, Table 1) is consistent with a facile reduction of the Ag(I) site, as found before for complexes **4**–**8** [28]^.^

**Table 1:** Cyclic voltammetry data^a^ for complexes [Ag(NO_3_)(L)] (1–3) and ligands L^I^-L^III^.

Complex	Potential			Ligand	Potential	
	${}^{I}E_{p}^{red}$	${}^{\mathrm{II}}E_{p}^{red}$	$E_{p}^{ox}$		${}^{I}E_{p}^{red}$	$E_{p}^{ox}$
**1**	0.13 ^b^	- 2.37	1.77	**L**^**I**^	—	1.60
**2**	0.14 ^b^	-1.69	1.63	**L**^**II**^	-1.84	1.62
**3**	0.049 ^b^	-1.50	1.34	**L**^**III**^	-1.72	1.31

^a^Values in Volt (±10 mV). Bu_4_NBF_4_/CH_3_CN (0.10M) used as electrolyte and [Fe(η^5^-C_5_H_5_)_2_]^0/+^ ($E_{1/2}^{ox}$ = 0.382 V *vs*. SCE) as internal reference.

^b^ Generates an adsorption wave.

The slightly lower Ag(I)→Ag(0) potential displayed by **3** compared to **1** and **2** (Table 1) indicates that there is a slightly higher electron density at the Ag(I) site due to electron withdrawing from the biphenyl bicamphor ligand (L^III^, Fig 1), as confirmed by the ligand based reduction at the complex occurring at a considerably higher potential than at the free ligand (Table 1). Such pattern renders the Ag(I) site slightly more electron-rich and thus more resistant to reduction, a fact that may be significant on the low antimicrobial activity displayed by **3** (see below). Complex **5** that displays a reduction potential ($E_{p}^{red}$ = 0.031 V) [28] similar to **3**, also displayed low antibacterial and antifungal activities. Data on redox properties of the complexes is still insufficient to draw further conclusions. However, it is worth to highlight that electron transfer could be responsible for formation of AgNPs upon interaction of the camphorimine Ag(I) complexes with *C*. *albicans* and the complexes have no antifungal activity (see below).

### Antimicrobial activity of the Ag(I) complexes

#### Antibacterial properties

The antibacterial properties of Ag(I) camphorimine complexes **1**–**3**, the ligands **L**^**I**^-**L**^**III**^ and AgNO_3_ were assessed based on the determination of the MIC values towards *S*. *aureus*, *B*. *contaminans*, *P*. *aeruginosa* and *E*. *coli* using the previously described methods and a modified Gompertz equation to estimate the MIC values [28,35]. No bacterial growth inhibition was detected for the ligands (**L**^**I**^-**L**^**III**^) in the range of concentrations: 10–1000 μg/mL (**L**^**II**^) and 2–150 μg/mL (**L**^**I**^ and **L**^**III**^) (Supporting information S1 Fig, S2 Fig, and S3 Fig). Lower solubility precluded the use of higher concentrations in the cases of **L**^**I**^ and **L**^**III**^. Low solubility of complex **3** also precluded studies using concentrations higher than 100 μg/mL. These results are consistent with the previously reported bactericidal activity of complexes **4**–**8** when used in concentrations equal or above the MIC [28].

The analysis of the antibacterial activity of complexes **1**, **2** and **3** towards *S*. *aureus*, *P*. *aeruginosa*, *B*. *contaminans* and *E*. *coli* (Supporting information S4 Fig, S5 Fig and S6 Fig) shows that complex **1** displays a MIC value lower than AgNO_3_ for *S*. *aureus* Newman. For the other bacterial strains complex **1** displays MIC values slightly higher than AgNO_3_ (Table 2). All the MIC values estimated for complexes **2** and **3** are considerably higher than those obtained for AgNO_3_ (Table 2). No colony forming units (CFUs) were detected for concentrations of complexes **1** and **2** equal or above the respective MIC values, demonstrating the bactericidal activity of the complexes. For complex **3,** no *P*. *aeruginosa* CFUs were detected for concentrations equal or above the estimated MIC value.

**Table 2:** MIC values (μg/mL)^a^ estimated for complexes [Ag(NO_3_)L] (L: L^I^, 1; L^II^, 2; L^III^, 3; L^IV^, 4; L^V^, 5; L^VI^, 6; L^VII^, 7; L^VIII^, 8).

Compound	*S*. *aureus* Newman	*B*. *contaminans* IST408	*P*. *aeruginosa* 477	*E*. *coli* ATCC 25922
**1**	66 ± 4.8	79 ± 4.4	56 ± 4.3	50 ± 1.0
**2**	183 ± 3.3	144 ± 1.0	121 ± 2.1	65 ± 1.5
**3**	˃ 100	˃ 100	86 ± 6.8	˃ 100
**4**^b^	73 ± 2.3	36 ± 2.5	19 ± 4.3	20 ± 1.3
**5** ^b^	118 ± 1.7	97 ± 1.2	68 ± 1.5	98 ± 1.3
**6** ^b^	119 ± 2.9	96 ± 1.6	105 ± 3.0	98 ± 1.3
**7** ^b^	95 ± 3.3	81 ± 4.5	39 ± 3.4	49 ± 1.5
**8** ^b^	259 ± 2.6	127 ± 4.6	138 ± 4.5	123 ± 4.0
AgNO_3_^b^	73 ± 1.9	74 ± 1.4	39 ± 2.0	47 ± 1.1

^**a**^ Towards *S*. *aureus*, *B*. *contaminans*, *P*. *aeruginosa* or *E*. *coli*. MIC values were estimated by fitting data from cultures optical density (measured at 640 nm) to a modified Gompertz equation (see experimental).

^b^ Data taken from reference [28].

#### Antifungal properties

The effect of the supplementation of the growth medium with Ag(I) complexes on the growth of the different yeast species under study is shown in S7A Fig to S7D Fig in Supporting information S7 Fig. The MIC_50_ values determined based on those results are compiled in Table 3. With the exception of **L**^**V**^, none of the ligands inhibited growth of the *Candida* spp tested (S8A Fig to S8D Fig in Supporting information S8 Fig). Nonetheless, the inhibitory effect of **L**^**v**^ (MIC_50_ within the range of 500–1000 μg/mL) was considerably smaller than the effect exerted by complex **5** (derived from it), this being particularly evident for *C*. *tropicalis* and *C*. *parapsilosis* (see S7C Fig and S8C Fig in Supporting information S7 Fig and S8 Fig). Within the range of concentrations tested none of the Ag(I) camphorimine complexes, nor AgNO_3_, showed efficacy in inhibiting growth of *C*. *albicans* (Table 3). This result was further confirmed by the observation that viability of *C*. *albicans* cells in the presence of 1000 μg/mL (the highest concentration used in the MIC assay) of all the compounds is identical to the one observed during cultivation in non-supplemented RPMI medium (results not shown).

**Table 3:** MIC values (μg/mL) for Ag(I) camphor complexes 1–8 and for AgNO_3_ against *C*. *albicans*, *C*. *glabrata*, *C*. *tropicalis* and *C*. *parapsilosis*, based on the EUCAST microdilution method [37].

Compound	*C*. *albicans*	*C*. *glabrata*	*C*. *tropicalis*	*C*. *parapsilosis*
**1**	> 1000	31.3	3.9	> 1000
**2**	> 1000	15.6	7.8	2.0
**3**	> 1000	62.5	31.3	15.6
**4**	> 1000	> 1000	> 1000	> 500
**5**	> 1000	125	3.9	2.0
**6**	> 1000	62.5	7.8	2.0
**7**	> 1000	31.3	3.9	2.0
**8**	> 1000	62.5	7.8	2.0
**AgNO**_**3**_	> 1000	15.6	3.9	2.0

Results in Table 3 show that the complexes display a degree of efficacy towards the *Candida* species ranging from non-active (**4**) to highly active (**2**). Consistent with the increased resilience of *C*. *glabrata* species to antimicrobials [38], the MIC values of all Ag(I) complexes obtained for this species are higher than those obtained for *C*. *tropicalis* or *C*. *parapsilosis* (Table 3). The inhibitory effect of the compounds on growth of *C*. *glabrata*, *C*. *parapsilosis* and *C*. *tropicalis* is fungistatic, since cellular viability at a concentration of the complexes equal to the MIC_50_ values was reduced but not fully abrogated (results not shown).

**Fig 3:**
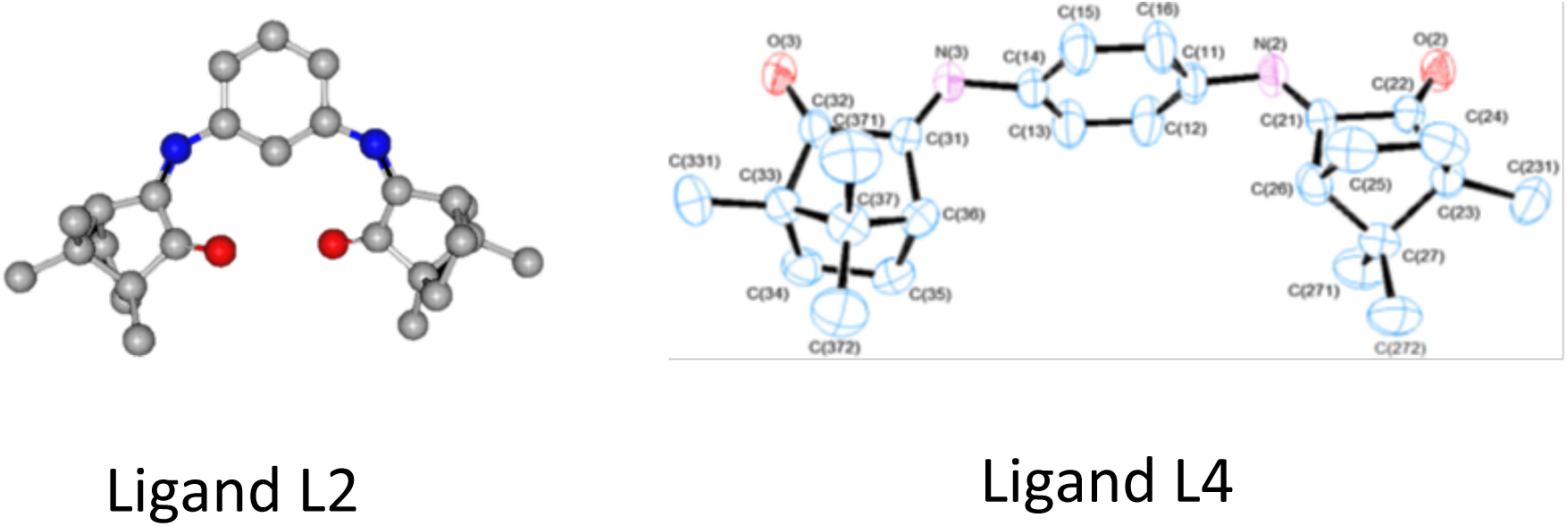
Spatial structures of ligands L2 and L4, evidencing that in complex 2 the Ag^+^metal ion finds a coordination geometry that conceivably provides a better protection, while in complex 4 it seems highly exposed to multiple interactions and deactivation due to reduction to Ag(0).

The results in this study reinforce the previously demonstrated potential of Ag(I) camphor complexes to be used as antimicrobials [28]. In this context, it is worth to highlight the high activity exhibited by the Ag(I) camphorimine complexes against the non-albicans *Candida* species *C*. *parapsilosis* and, to a lesser extent, against *C*. *tropicalis*. The incidence of infections caused by these species has been increasing due to increase of resistance to currently used antifungals and to their remarkable ability to thrive in indwelling medical devices [39]. It is interesting to observe that the camphorimine Ag(I) complexes **1**–**8** appear to exert specific and distinct effects against bacteria and *Candida* cells. For example, complex **4** is highly active against bacteria but less effective in inhibiting *Candida* spp. growth, while complex **2** is highly active against the *Candida* spp. (with MIC values identical to those estimated for AgNO_3_) and one of the less active compounds against the set of bacterial strains tested. Complexes prepared with ligands L**2** and L**4** essentially differ on the aromatic spacer binding the two camphor moieties at *meta* or *para* positions, in the ligands (Fig 1). However, this apparently small difference enforces steric modifications, that according to the results obtained drive the interaction with the biological receptors and consequently to the antimicrobial activities of the complexes. In a former work, camphorimine compounds L2 and L4 were used as ligands to prepare Cu(I) complexes and their anti-proliferative activity against the cancer cell line HT29 was studied [32]. Both complexes displayed high anti-proliferative activity, being the activity of the Cu(I) complex with ligand **L2** higher compared to **L4**. This is consistent with the higher antifungal activity of Ag^+^ complexed with **L2** compared to **L4**. The geometry of the bicamphor imine ligands **L2** and **L4** is highly different (Fig 3). When complexed with **L2**, the metal Ag^+^ finds a coordination geometry that conceivably provides it a better protection, while when complexed with **L4** it is more exposed and prone to multiple interactions. Since the activity of metal complexes with ligand **L2** towards eukaryotic cells is higher than when complexed with **L4**, we speculate that both the geometry of the complexes and the distinct lipid and protein content of eukaryotic and prokaryotic cell membranes might determine the distinct specificity of the compounds.

Another relevant point from this study is that complexes for which a MIC value could be determined showed to be bactericidal (when used in concentrations equal or above the MIC) while they are fungistatic. Such behaviour suggests that there are different biological targets in the prokaryotic and eukaryotic cells or, that complexes penetrate fungal and bacterial cells through different pathways, their structural arrangements finely-tuning the reactivity. There are marked differences between the structure of the cell envelope of the tested bacterial strains and *Candida* species which could sustain different cellular permeability to the different Ag(I) complexes. Further studies are required to deepen the understanding of the mechanisms underlying the specificity of the inhibitory effects exerted by Ag(I) complexes on the growth of *Candida* spp. and of bacteria.

Although the antibacterial and antifungal activities of the Ag(I) camphorimine complexes indicate their potential as antimicrobials, their feasible use in clinical practice requires the evaluation of their toxicity against mammalian cells.

#### AgNPs from Ag(I) camphor complexes synthesized by *C*. *albicans*

**Fig 4:**
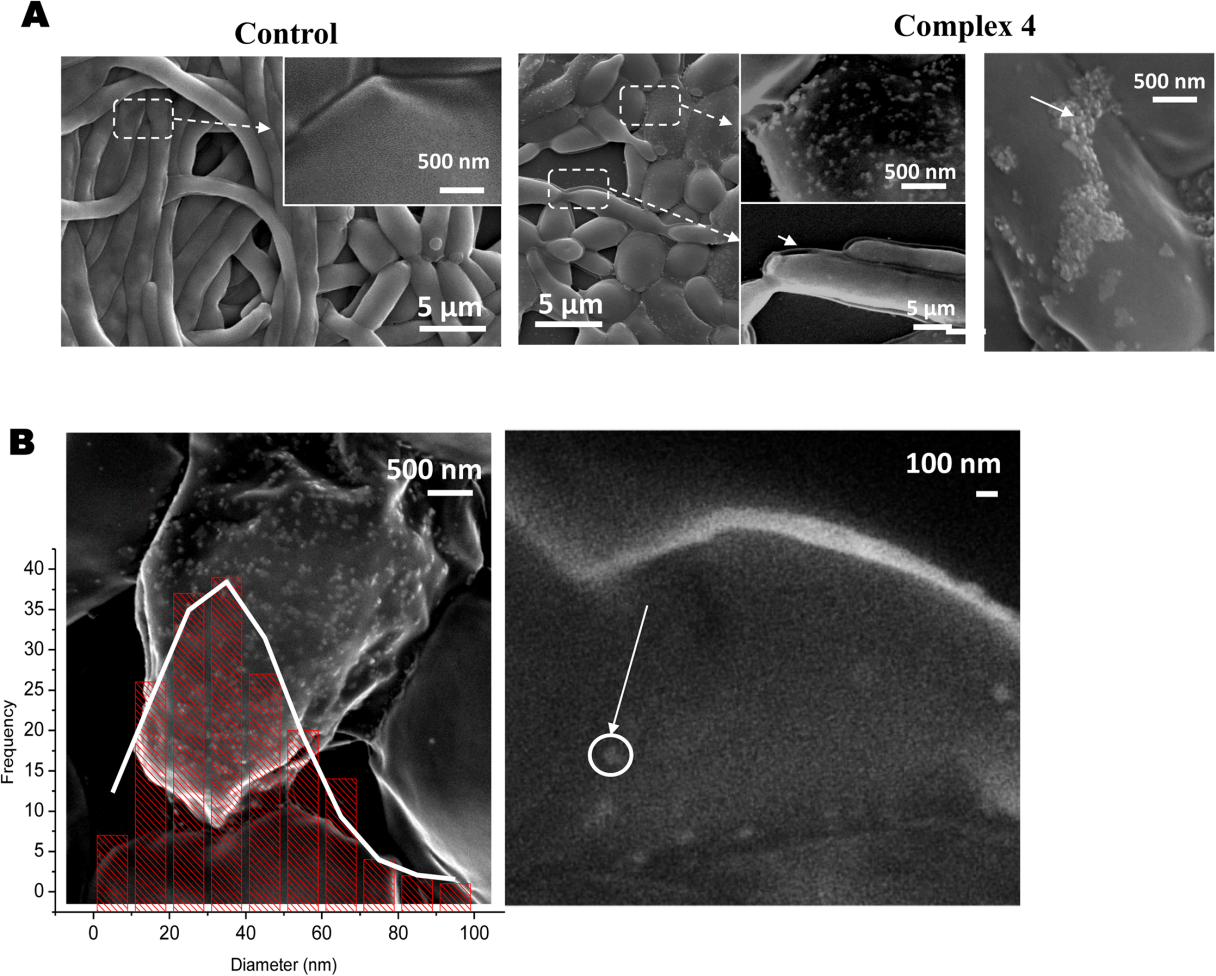
Microscopy images obtained by SEM of *C*. *albicans* cells SC5314 when cultivated for 24h in: A1- RPMI growth medium (control); A2- RPMI growth medium supplemented with 125 μg/mL of 4. Panel A2 shows a magnification of the image evidencing the occurrence of modifications on the cell surface; B- size distribution of the small nanoparticles found at the cell surface of *C*. *albicans*.

An intriguing point on the study of the antifungal properties of the Ag(I) camphorimine complexes **1**–**8** is their generalized lack of effect in inhibiting growth of *C*. *albicans*. To further examine this issue, cellular suspensions of *C*. *albicans* were incubated in the presence or absence of complexes **1** and **4** and afterwards examined by scanning electronic microscopy (SEM). The SEM pictures evidenced significant morphological alterations in C. *albicans* cells exposed to complexes **1** and **4** and cells cultivated in the absence of the complexes. Cells exposed to complexes **1** and **4** showed a marked reduction of the hyphae formed and alterations at the cell surface, as displayed in Fig 4.

Small protuberances (at the nanoscale level—Fig 4A2) were detected on the cell surface of *C*. *albicans* upon exposure to Ag(I) complexes **1** and **4**. The calculated size (33±18 nm) and subsequent elemental analysis by EDS confirmed these nanoparticles to be silver nanoparticles (Fig 3B and results not shown). It is important to stress that no AgNPs were detected on samples of complexes **1** and **4** that had not been exposed to the *C*. *albicans* (Supporting information S9 Fig). These observations support Ag(I)→Ag(0) reduction by *C*. *albicans* with concomitant formation of silver nanoparticles (AgNP*s*). Consistently, AgNPs are known to inhibit formation of hyphae and to affect the cell wall structure of *C*. *albicans* [16,17], two effects that we have observed to occur in our assays (Fig 4A and 4B). AgNPs have a milder antifungal activity [17,40] than Ag^+^ and thus the ability of *C*. *albicans* to convert the camphorimine Ag(I) complexes into AgNPs is likely to be one of the causes for the lack of activity of complexes **1**–**8** against *C*. *albicans*.

Reports of fungal-mediated synthesis of AgNPs from Ag^+^ have been published before [41,42]. However, to the best of our knowledge, this is the first time that the widely used *C*. *albicans* SC5314 reference strain is reported to synthesize silver nanoparticles.

The ability of *C*. *albicans* cells to prompt reduction of Ag(I) to Ag(0) while the other *Candida* spp. used in this study do not suggest further studies (out of the scope of this work). The mechanism of formation of AgNPs promoted by fungal cells remains to be elucidated, although it has been suggested that it involves the initial adsorption of Ag^+^ ions to the surface of the fungal cells promoted by electrostatic interactions between carboxylate groups (negatively charged) at the cell wall and the Ag^+^ ions (positively charged). Electron transfer from the enzymes to the silver ions would then be responsible for their reduction leading to formation of silver particles [17]. In the case of camphorimine complexes, it was shown by cyclic voltammetry (see above) that Ag(I)→Ag(0) reduction occur at potentials 0.14–0.049 V (Table 1) accessible to enzymes. Formation of AgNPs suggests that *C*. *albicans* harbors at least one protein able to trigger electron transfer and promote this reduction process. The other *Candida* species conceivably do not have at their cell surface proteins analogous to those in *C*. *albicans* able to mediate Ag^+^ reduction, thus no formation of AgNPs is detected. An interesting strategy to sensitize *C*. *albicans* cells against Ag-based compounds could be to target the activity of these enzymes mediating Ag-reduction.

### Conclusions

All the camphorimine [Ag(NO_3_)L] complexes **1**–**8** assessed for antimicrobial properties display bactericidal activity against *B*. *contaminans* and the bacterial species of the ESKAPE group *S*. *aureus*, *P*. *aeruginosa*, and *E*. *coli*. The low MIC values observed for some of the complexes suggest their potential use as as alternatives to the existing antibiotics. In addition to their bactericidal properties, the complexes (except complex **4**) also display fungistatic activity against the three pathogenic *Candida* species *C*. *tropicalis*, *C*. *parapsilosis*, and *C*. *glabrata*. The camphorimine Ag(I) complexes are especially active against *C*. *parapsilosis*, a recognized emergent agent of fungal infections. The fact that complexes behave as bactericidal but fungistatic suggests that they have different targets and/or mechanisms of action in prokaryotic and eukaryotic cells.

None of the complexes is active against *C*. *albicans* SC5314 which is able to promote the reduction of the Ag(I) site forming AgNPs as confirmed by SEM.

Since the molecular formulae of the complexes **1**–**8** essentially differ on the ligand L, their distinct antimicrobial properties have to be based on the characteristic of the camphorimine ligands. In fact, complexes **2** and **4** that just differ on the geometry of the aromatic spacer between the two camphor ligands, display opposite properties, *i*.*e*. complex **2** displays low antibacterial and high antifungal properties while complex **4** displays high antibacterial and low antifungal activity. Electron delocalization may one of the parameters responsible for increases antifungal activity in complexes **1**, **3**, **4** and **5**, while no such trend is detected for the antibacterial properties. Further efforts are necessary to fully understand the processes underlying the antimicrobial activities which may be relevant to the development of Ag(I) camphor-derived chemicals, including AgNPs as anti-*Candida* agents.

Overall, the results now reported show that antifungal and antibacterial properties combine in Ag(I) camphorimine complexes which have potential to be used as alternative therapeutic compounds for the development of new antimicrobials against clinically relevant pathogenic bacterial and *Candida* species.

## Supporting information

S1 Fig Antibacterial activity of ligand L^I^ towards *S*. *aureus*, *B*. *contaminans*, *P*. *aeruginosa* or *E*. *coli*. (PPT)

S2 Fig Antibacterial activity of ligand L^II^ towards *S*. *aureus*, *B*. *contaminans*, *P*. *aeruginosa* or *E*. *coli*. (PPT)

S3 Fig Antibacterial activity of ligand L^III^ towards *S*. *aureus*, *B*. *contaminans*, *P*. *aeruginosa* or *E*. *coli*. (PPT)

S4 Fig Antibacterial activity of complex 1 towards *S*. *aureus*, *B*. *contaminans*, *P*. *aeruginosa* or *E*. *coli*. (PPT)

S5 Fig Antibacterial activity of complex 2 towards *S*. *aureus*, *B*. *contaminans*, *P*. *aeruginosa* or *E*. *coli*. (PPT)

S6 Fig Antibacterial activity of complex 3 towards *S*. *aureus*, *B*. *contaminans*, *P*. *aeruginosa* or *E*. *coli*. (PPT)

S7 Fig Activity of complexes 1 to 8 against *C*. *albicans*, *C*. *glabrata*, *C*. *tropicalis* and *C*. *parapsilosis*. (PPTX)

S8 Fig Activity of ligands L^I^ to L^VIII^ against *C*. *albicans*, *C*. *glabrata*, *C*. *tropicalis* and *C*. *parapsilosis* (PPTX)

S9 Fig Structure of complexes 1 and 4 as observed by Scanning Electron Microscopy. (TIF)
