# Supplementary material for: Ag(I) camphorimine complexes with antimicrobial activity towards clinically important bacteria and species of the Candida genus
Source: PLoS One. 2017 May 9;12(5):e0177355. doi: 10.1371/journal.pone.0177355 (PMC5423651; doi:10.1371/journal.pone.0177355)
Supplement: S1 Fig — (PPT) [file pone.0177355.s001.ppt]

## Slide 1
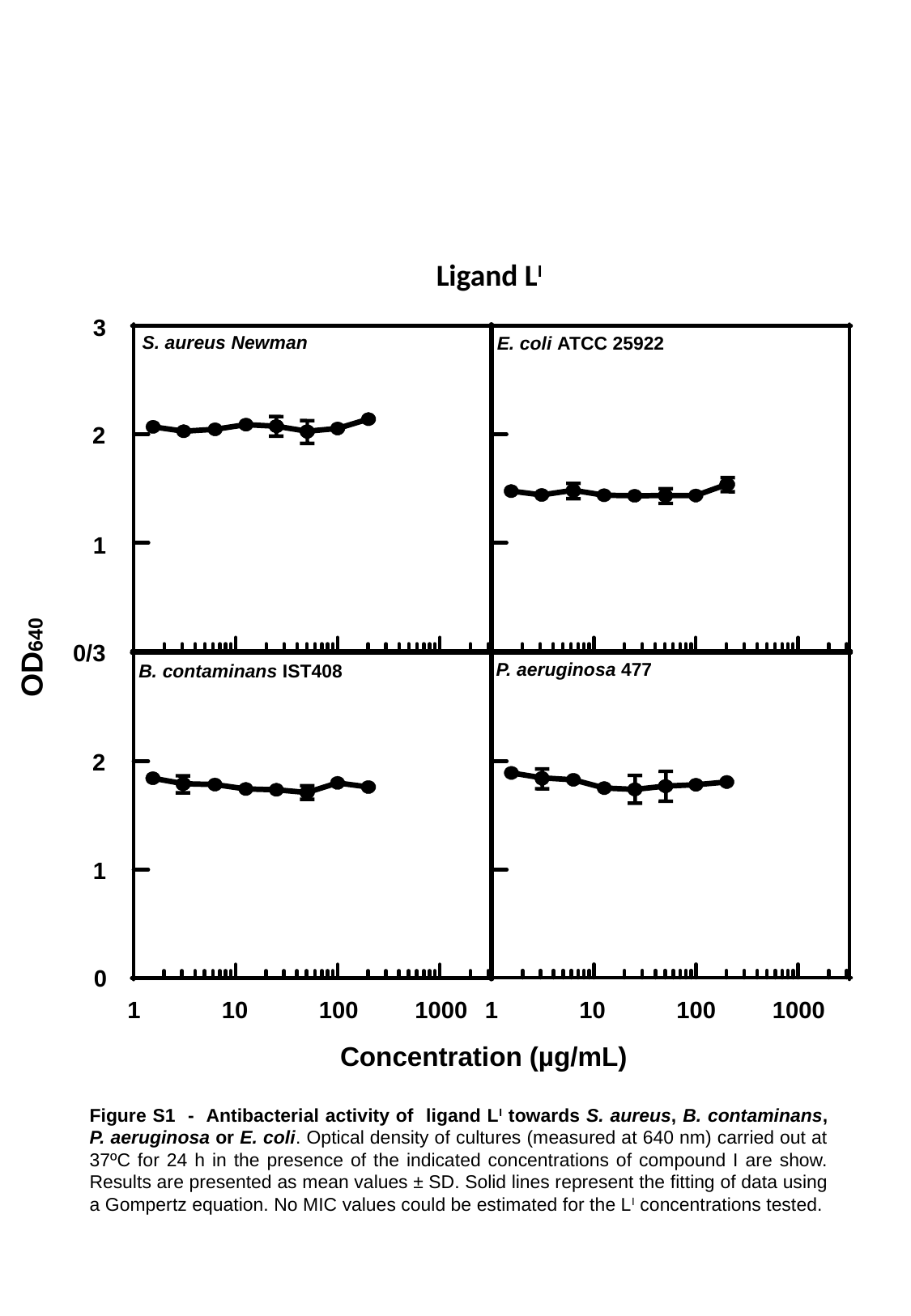

Ligand LI
3
1
2
S. aureus Newman
E. coli ATCC 25922
0/3
1
0
2
OD640
P. aeruginosa 477
B. contaminans IST408
1
10
1000
100
1
10
1000
100
Concentration (µg/mL)
Figure S1 - Antibacterial activity of ligand LI towards S. aureus, B. contaminans, P. aeruginosa or E. coli. Optical density of cultures (measured at 640 nm) carried out at 37ºC for 24 h in the presence of the indicated concentrations of compound I are show. Results are presented as mean values ± SD. Solid lines represent the fitting of data using a Gompertz equation. No MIC values could be estimated for the LI concentrations tested.
