# Supplementary material for: Ag(I) camphorimine complexes with antimicrobial activity towards clinically important bacteria and species of the Candida genus
Source: PLoS One. 2017 May 9;12(5):e0177355. doi: 10.1371/journal.pone.0177355 (PMC5423651; doi:10.1371/journal.pone.0177355)
Supplement: S5 Fig — (PPT) [file pone.0177355.s005.ppt]

## Slide 1
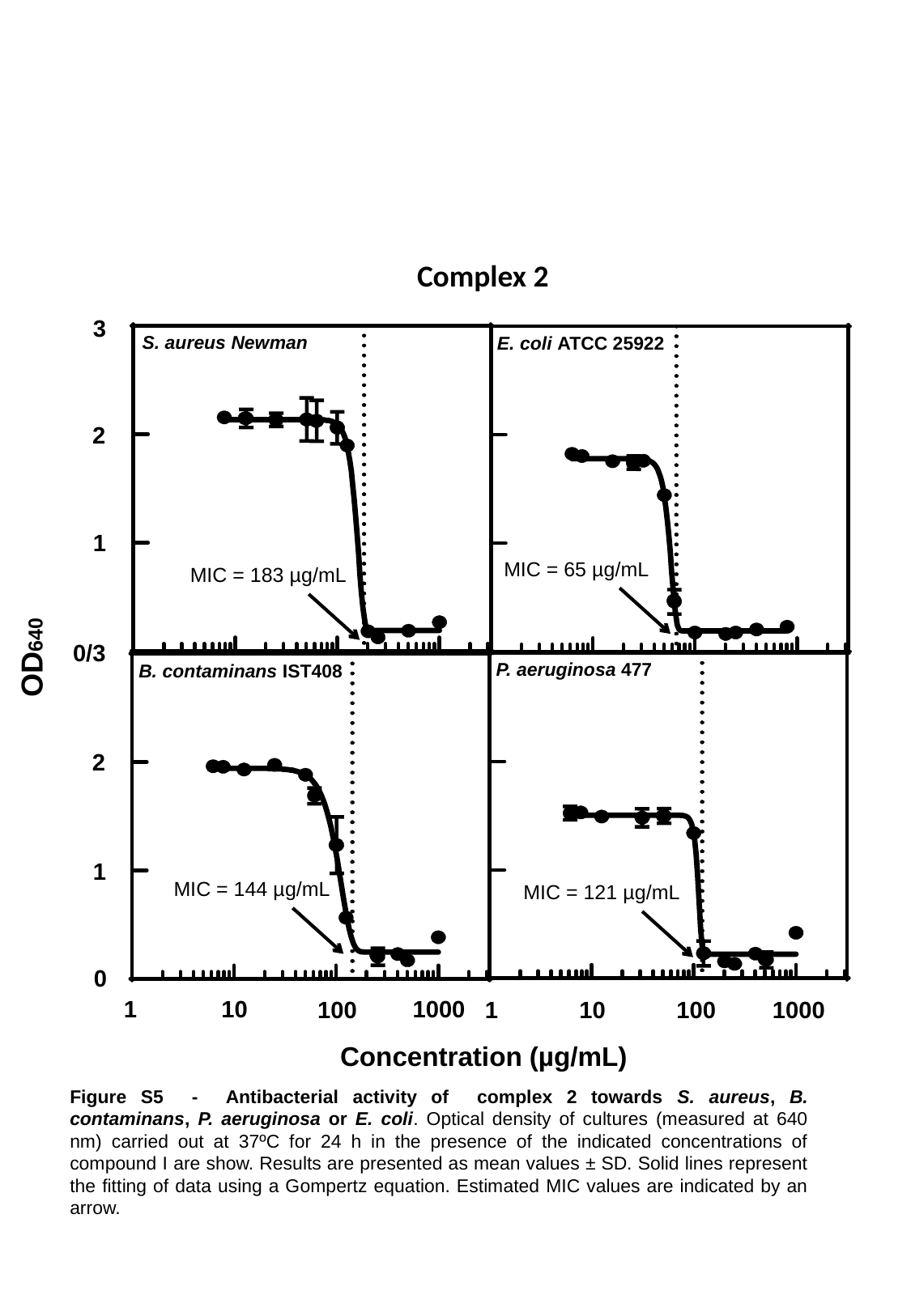

Complex 2
3
1
2
S. aureus Newman
MIC = 183 µg/mL
E. coli ATCC 25922
MIC = 65 µg/mL
0/3
1
0
2
OD640
B. contaminans IST408
MIC = 144 µg/mL
1
10
1000
100
MIC = 121 µg/mL
P. aeruginosa 477
1
10
1000
100
Concentration (µg/mL)
Figure S5 - Antibacterial activity of complex 2 towards S. aureus, B. contaminans, P. aeruginosa or E. coli. Optical density of cultures (measured at 640 nm) carried out at 37ºC for 24 h in the presence of the indicated concentrations of compound I are show. Results are presented as mean values ± SD. Solid lines represent the fitting of data using a Gompertz equation. Estimated MIC values are indicated by an arrow.
