# Supplementary material for: Ag(I) camphorimine complexes with antimicrobial activity towards clinically important bacteria and species of the Candida genus
Source: PLoS One. 2017 May 9;12(5):e0177355. doi: 10.1371/journal.pone.0177355 (PMC5423651; doi:10.1371/journal.pone.0177355)
Supplement: S7 Fig — (PPTX) [file pone.0177355.s007.pptx]

## Slide 1
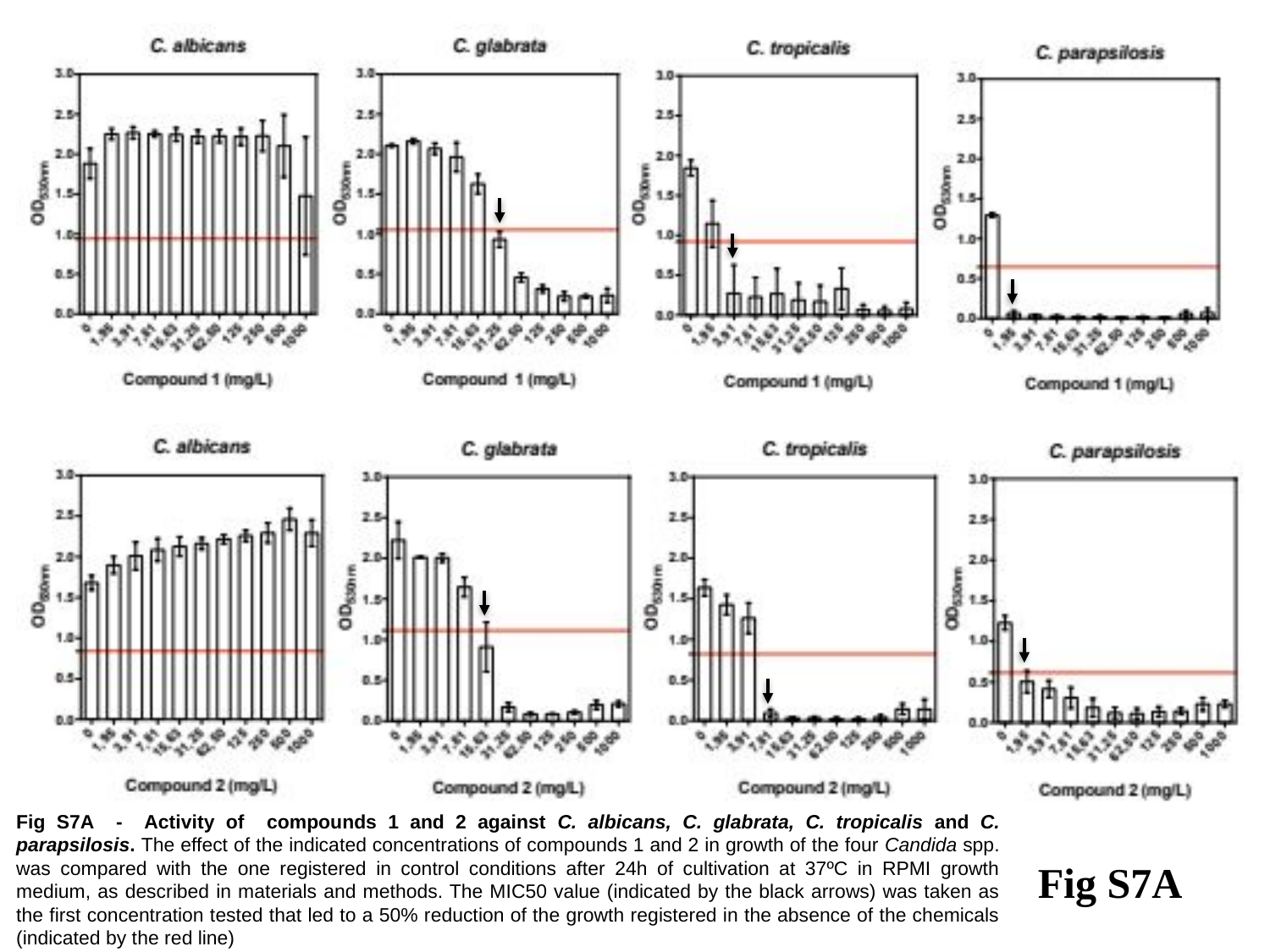

Fig S7A - Activity of compounds 1 and 2 against C. albicans, C. glabrata, C. tropicalis and C. parapsilosis. The effect of the indicated concentrations of compounds 1 and 2 in growth of the four Candida spp. was compared with the one registered in control conditions after 24h of cultivation at 37ºC in RPMI growth medium, as described in materials and methods. The MIC50 value (indicated by the black arrows) was taken as the first concentration tested that led to a 50% reduction of the growth registered in the absence of the chemicals (indicated by the red line)
Fig S7A

## Slide 2
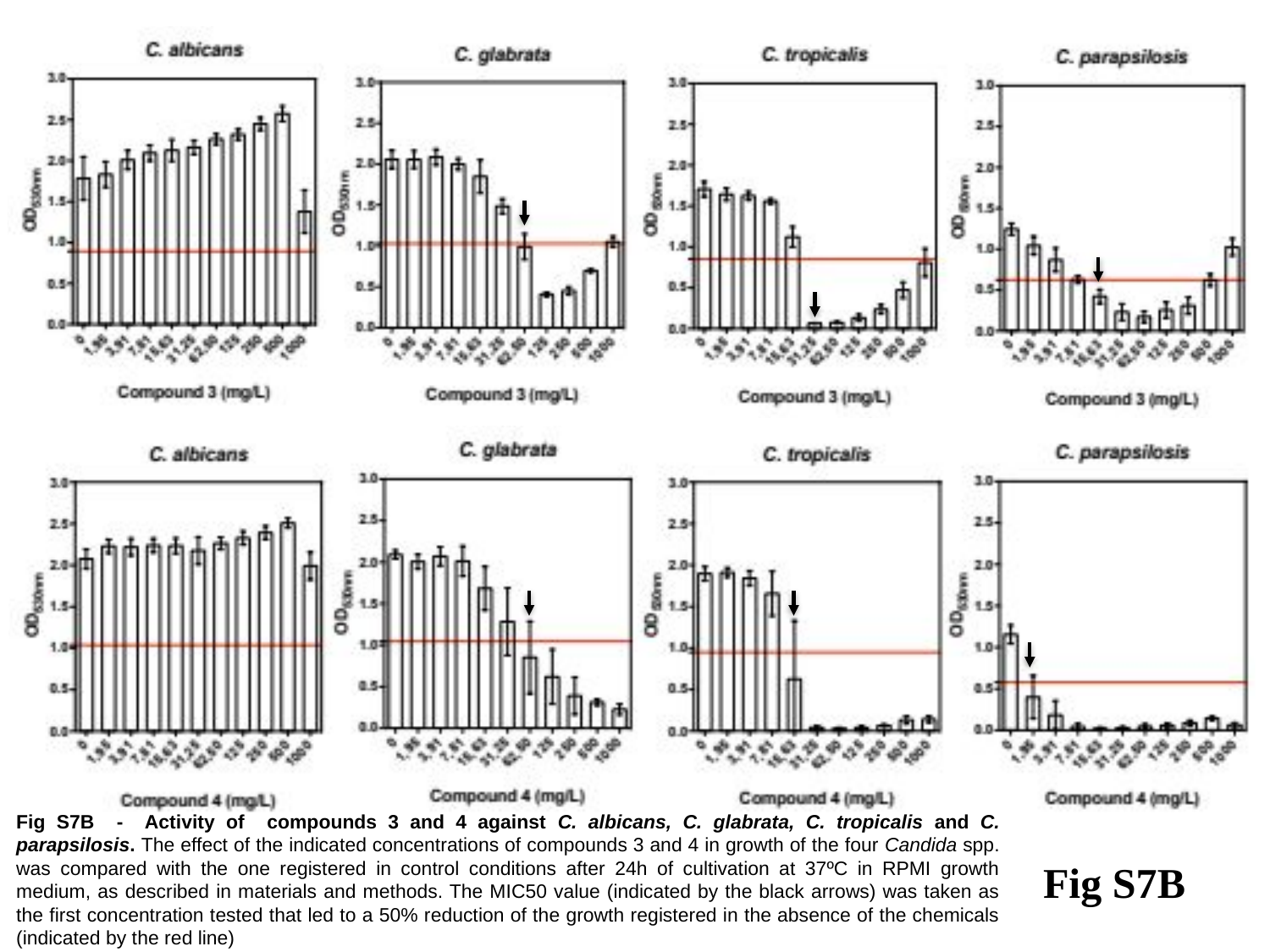

Fig S7B - Activity of compounds 3 and 4 against C. albicans, C. glabrata, C. tropicalis and C. parapsilosis. The effect of the indicated concentrations of compounds 3 and 4 in growth of the four Candida spp. was compared with the one registered in control conditions after 24h of cultivation at 37ºC in RPMI growth medium, as described in materials and methods. The MIC50 value (indicated by the black arrows) was taken as the first concentration tested that led to a 50% reduction of the growth registered in the absence of the chemicals (indicated by the red line)
Fig S7B

## Slide 3
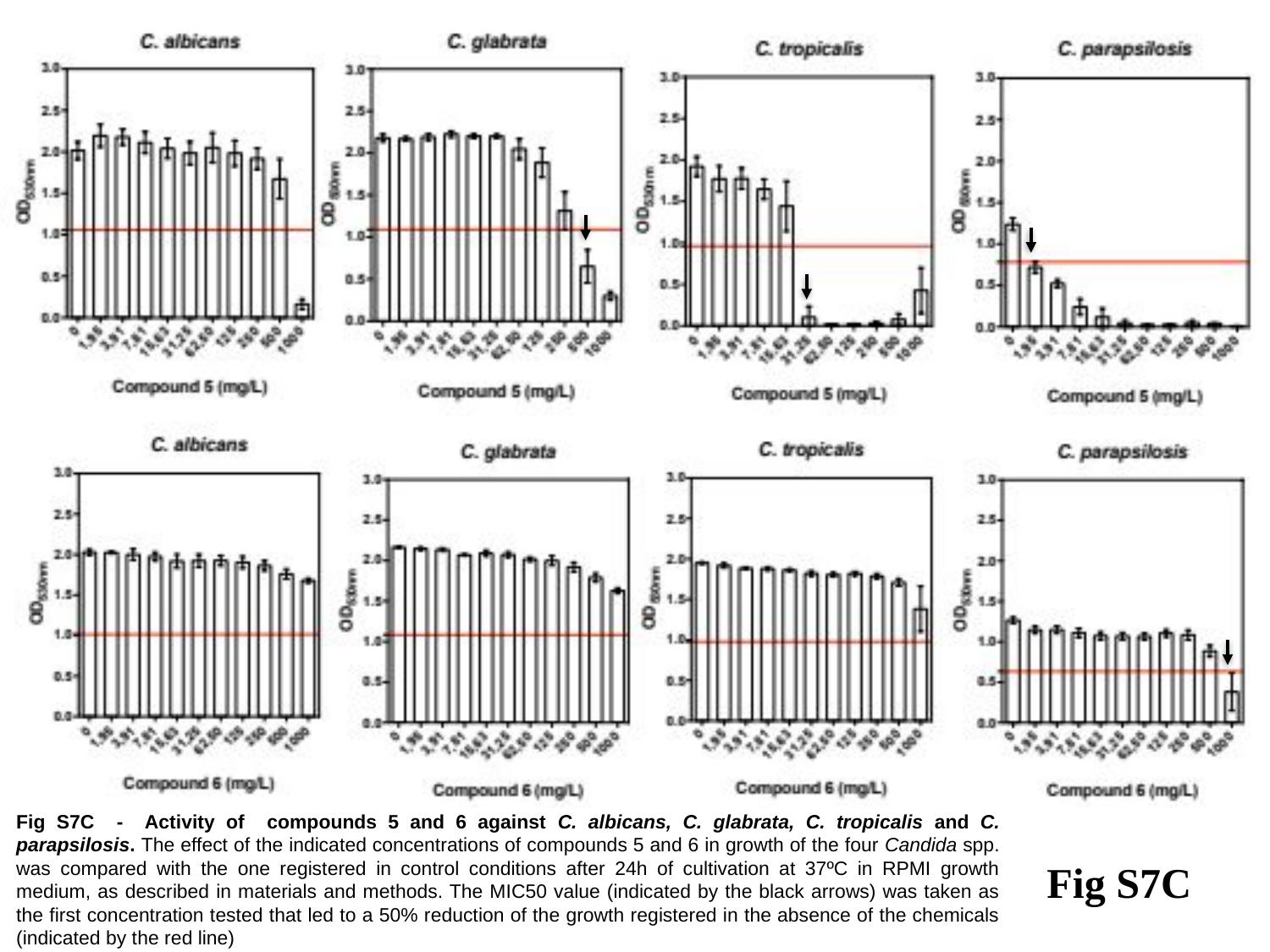

Fig S7C - Activity of compounds 5 and 6 against C. albicans, C. glabrata, C. tropicalis and C. parapsilosis. The effect of the indicated concentrations of compounds 5 and 6 in growth of the four Candida spp. was compared with the one registered in control conditions after 24h of cultivation at 37ºC in RPMI growth medium, as described in materials and methods. The MIC50 value (indicated by the black arrows) was taken as the first concentration tested that led to a 50% reduction of the growth registered in the absence of the chemicals (indicated by the red line)
Fig S7C

## Slide 4
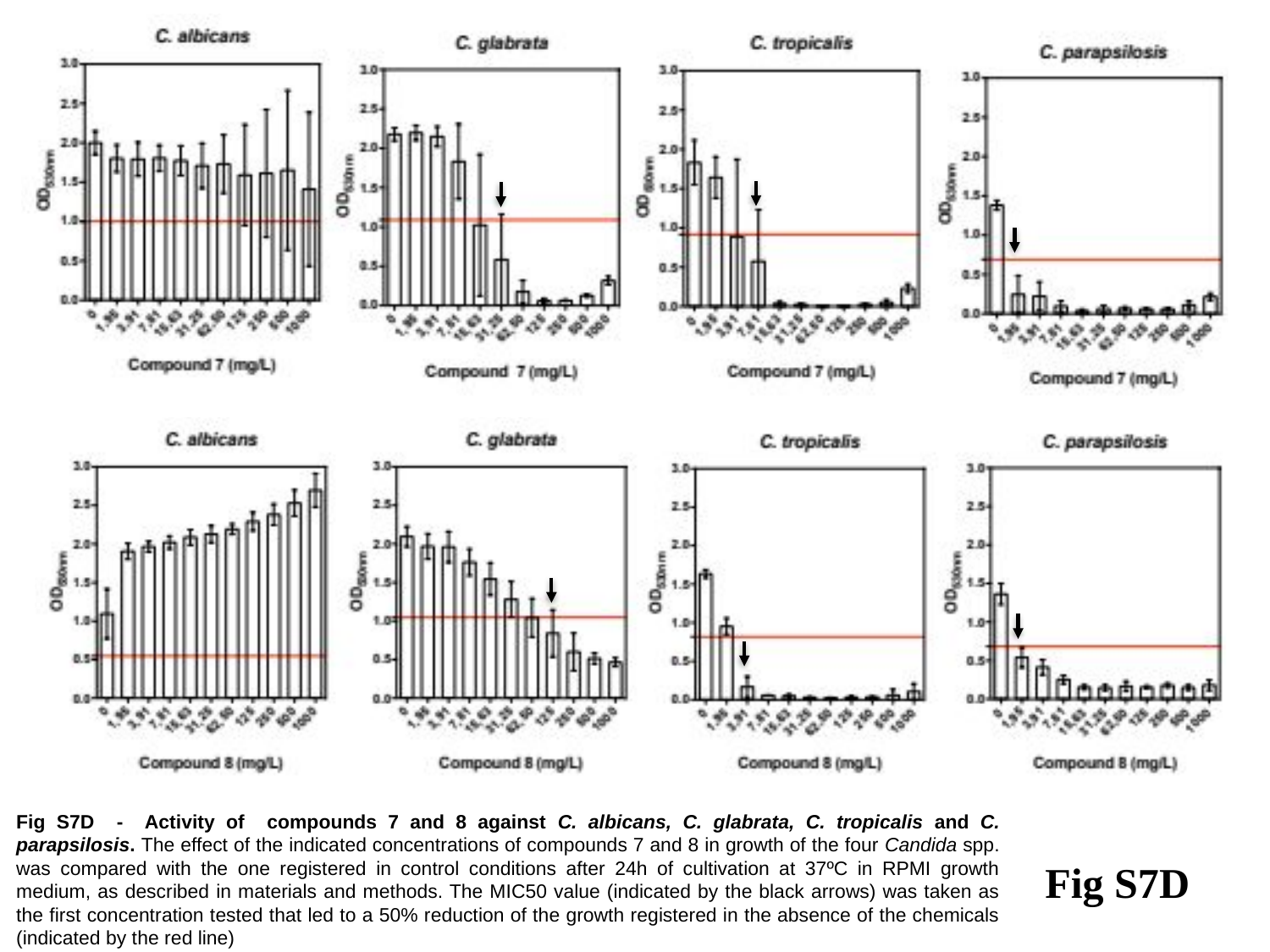

Fig S7D - Activity of compounds 7 and 8 against C. albicans, C. glabrata, C. tropicalis and C. parapsilosis. The effect of the indicated concentrations of compounds 7 and 8 in growth of the four Candida spp. was compared with the one registered in control conditions after 24h of cultivation at 37ºC in RPMI growth medium, as described in materials and methods. The MIC50 value (indicated by the black arrows) was taken as the first concentration tested that led to a 50% reduction of the growth registered in the absence of the chemicals (indicated by the red line)
Fig S7D
