# Supplementary material for: Ag(I) camphorimine complexes with antimicrobial activity towards clinically important bacteria and species of the Candida genus
Source: PLoS One. 2017 May 9;12(5):e0177355. doi: 10.1371/journal.pone.0177355 (PMC5423651; doi:10.1371/journal.pone.0177355)
Supplement: S8 Fig — (PPTX) [file pone.0177355.s008.pptx]

## Slide 1
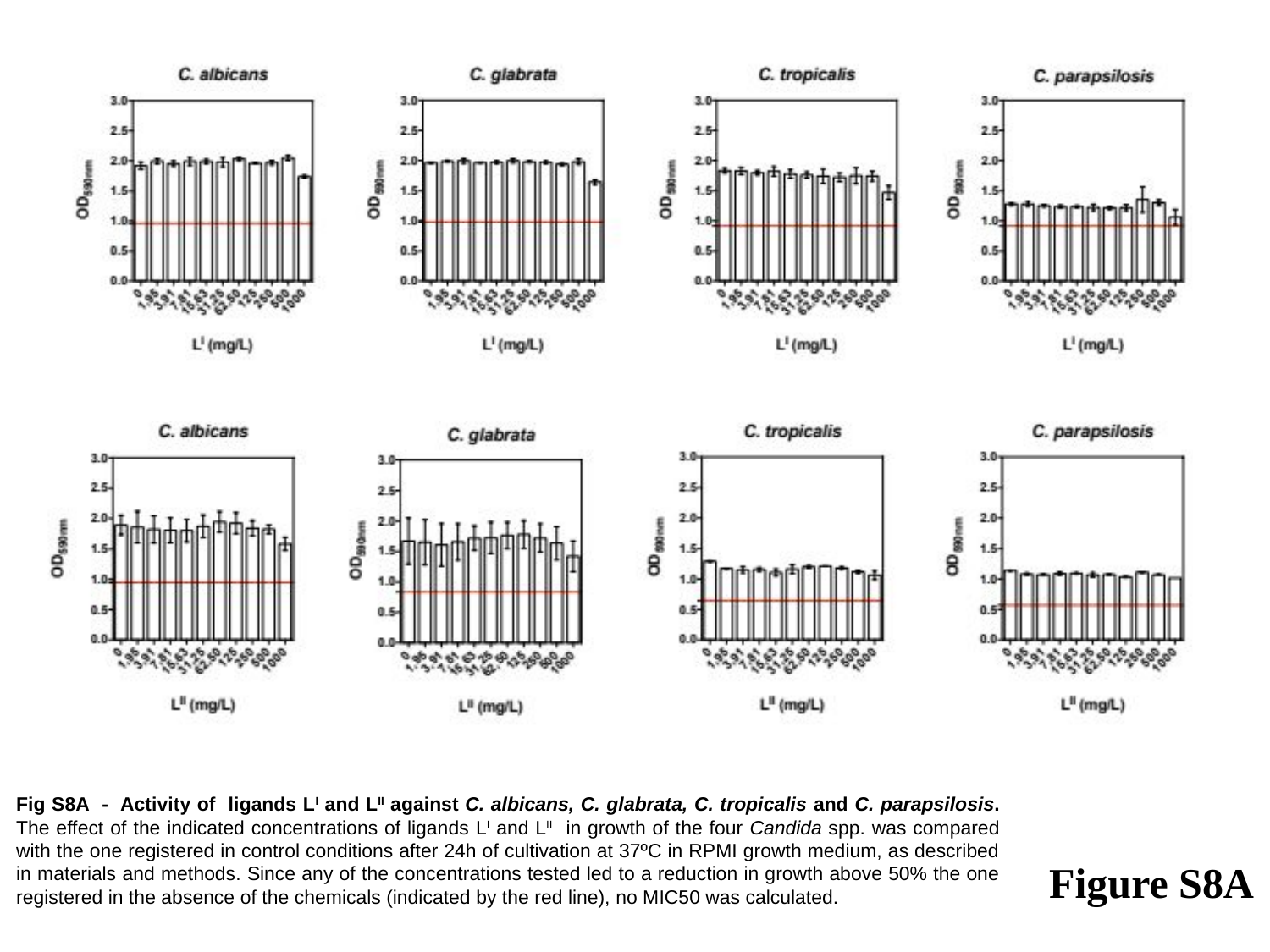

Fig S8A - Activity of ligands LI and LII against C. albicans, C. glabrata, C. tropicalis and C. parapsilosis. The effect of the indicated concentrations of ligands LI and LII in growth of the four Candida spp. was compared with the one registered in control conditions after 24h of cultivation at 37ºC in RPMI growth medium, as described in materials and methods. Since any of the concentrations tested led to a reduction in growth above 50% the one registered in the absence of the chemicals (indicated by the red line), no MIC50 was calculated.
Figure S8A

## Slide 2
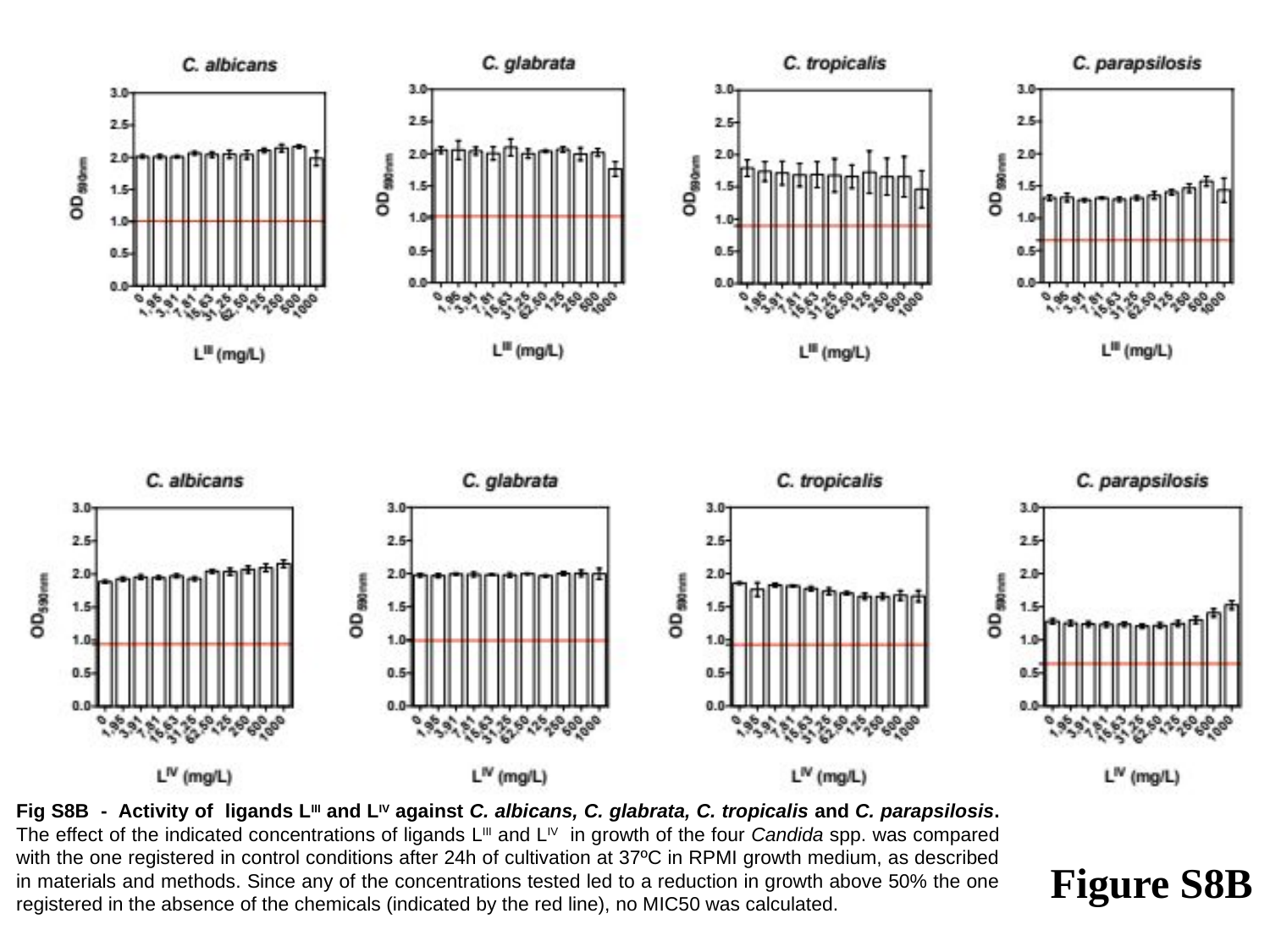

Fig S8B - Activity of ligands LIII and LIV against C. albicans, C. glabrata, C. tropicalis and C. parapsilosis. The effect of the indicated concentrations of ligands LIII and LIV in growth of the four Candida spp. was compared with the one registered in control conditions after 24h of cultivation at 37ºC in RPMI growth medium, as described in materials and methods. Since any of the concentrations tested led to a reduction in growth above 50% the one registered in the absence of the chemicals (indicated by the red line), no MIC50 was calculated.
Figure S8B

## Slide 3
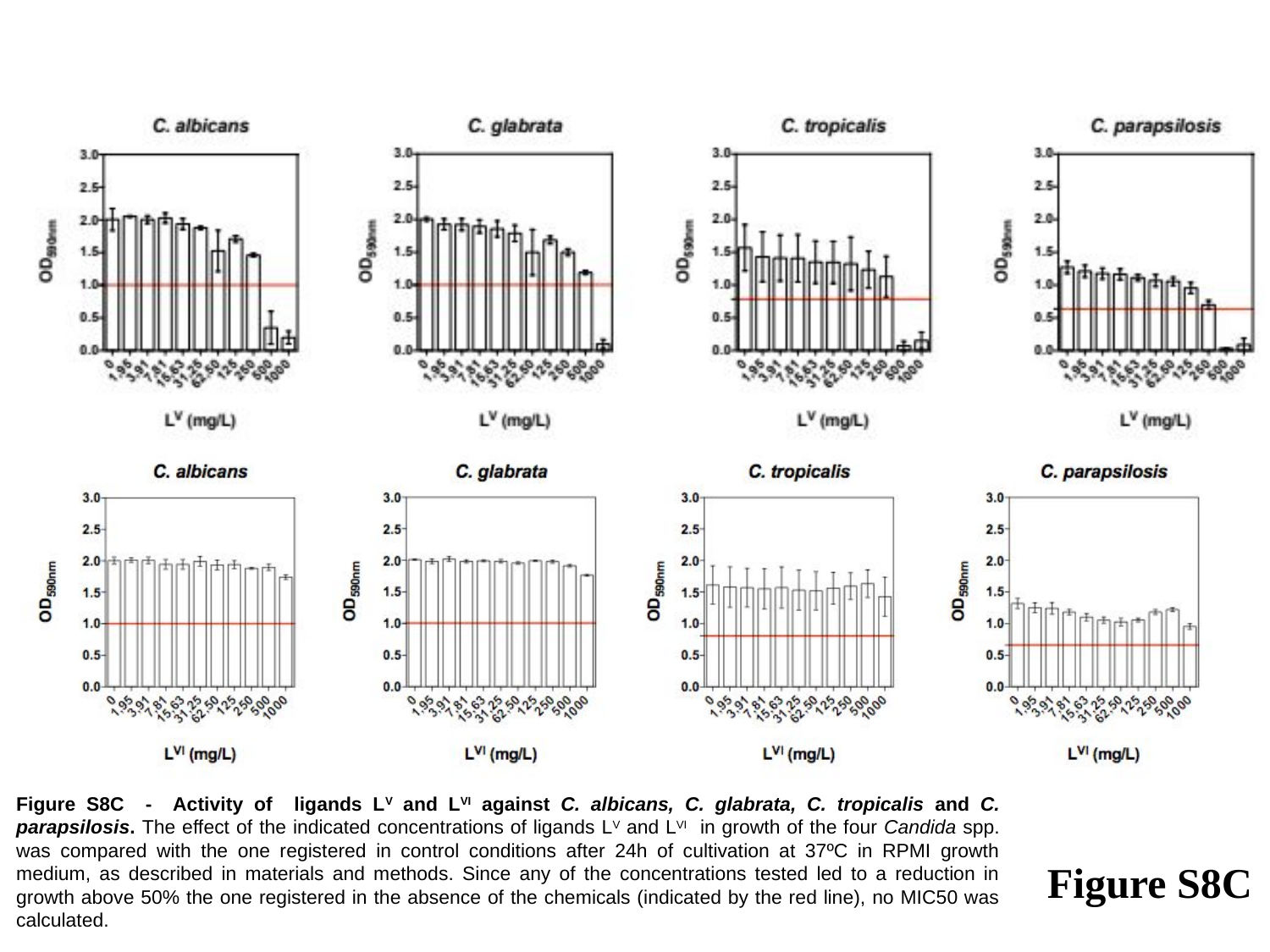

Figure S8C - Activity of ligands LV and LVI against C. albicans, C. glabrata, C. tropicalis and C. parapsilosis. The effect of the indicated concentrations of ligands LV and LVI in growth of the four Candida spp. was compared with the one registered in control conditions after 24h of cultivation at 37ºC in RPMI growth medium, as described in materials and methods. Since any of the concentrations tested led to a reduction in growth above 50% the one registered in the absence of the chemicals (indicated by the red line), no MIC50 was calculated.
Figure S8C

## Slide 4
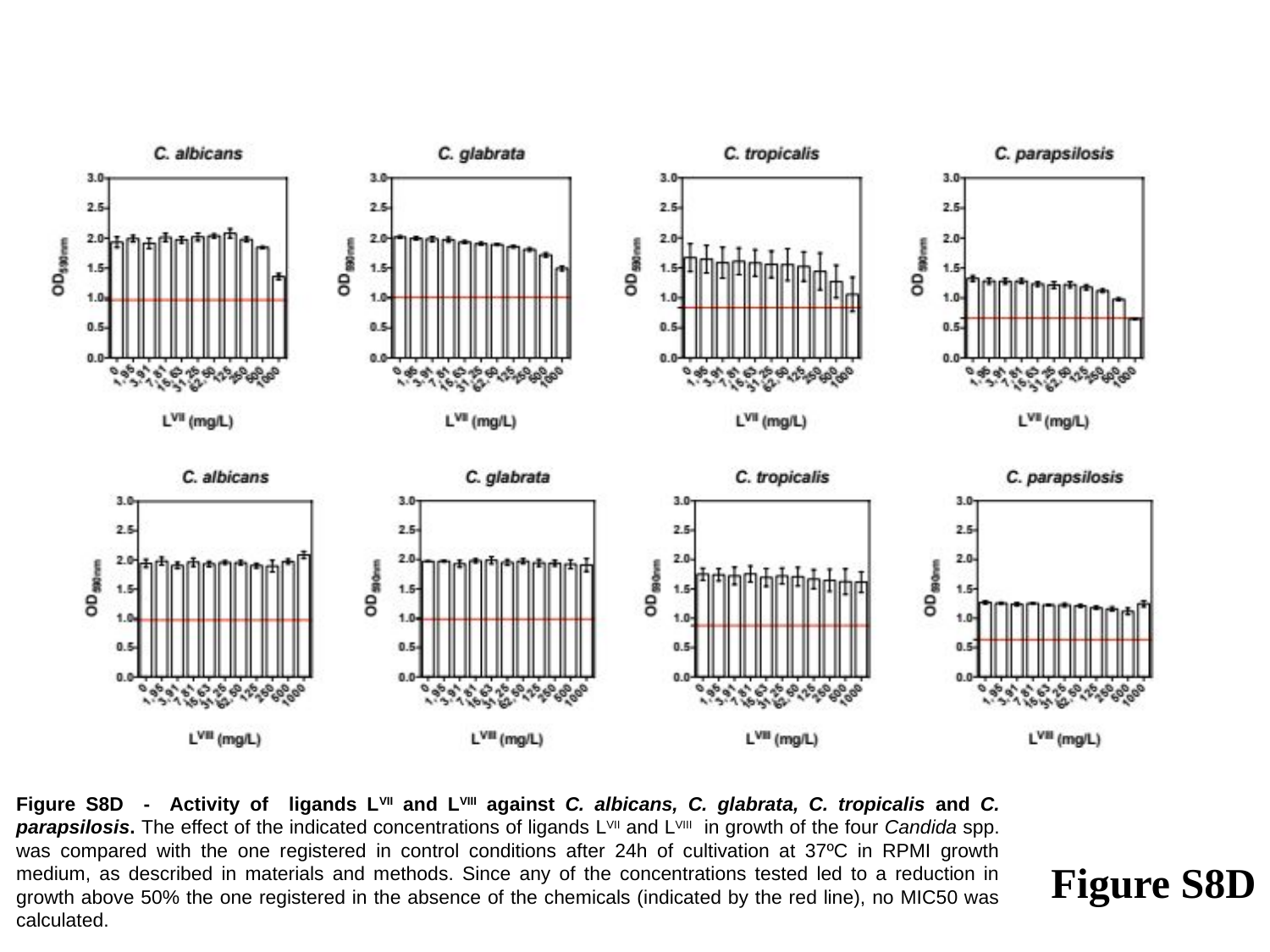

Figure S8D - Activity of ligands LVII and LVIII against C. albicans, C. glabrata, C. tropicalis and C. parapsilosis. The effect of the indicated concentrations of ligands LVII and LVIII in growth of the four Candida spp. was compared with the one registered in control conditions after 24h of cultivation at 37ºC in RPMI growth medium, as described in materials and methods. Since any of the concentrations tested led to a reduction in growth above 50% the one registered in the absence of the chemicals (indicated by the red line), no MIC50 was calculated.
Figure S8D
